# Atypical changes in DRG neuron excitability and complex pain phenotype associated with a Na_v_1.7 mutation that massively hyperpolarizes activation

**DOI:** 10.1038/s41598-018-20221-7

**Published:** 2018-01-29

**Authors:** Jianying Huang, Malgorzata A. Mis, Brian Tanaka, Talia Adi, Mark Estacion, Shujun Liu, Suellen Walker, Sulayman D. Dib-Hajj, Stephen G. Waxman

**Affiliations:** 1 Department of Neurology and Center for Neuroscience and Regeneration Research, Yale University School of Medicine, New Haven, CT USA 06510; 2 Rehabilitation Research Center, Veterans Affairs Connecticut Healthcare System, West Haven, CT USA 06516; 3 Developmental Neurosciences Program, Department of Anaesthesia and Pain Medicine, UCL Great Ormond Street Hospital, London, WC1N 1EH UK

## Abstract

Sodium channel Na_v_1.7 plays a central role in pain-signaling: gain-of-function Na_v_1.7 mutations usually cause severe pain and loss-of-function mutations produce insensitivity to pain. The Na_v_1.7 I234T gain-of-function mutation, however, is linked to a dual clinical presentation of episodic pain, together with absence of pain following fractures, and corneal anesthesia. How a Na_v_1.7 mutation that produces gain-of-function at the channel level causes clinical loss-of-function has remained enigmatic. We show by current-clamp that expression of I234T in dorsal root ganglion (DRG) neurons produces a range of membrane depolarizations including a massive shift to >−40 mV that reduces excitability in a small number of neurons. Dynamic-clamp permitted us to mimic the heterozygous condition via replacement of 50% endogenous wild-type Na_v_1.7 channels by I234T, and confirmed that the I234T conductance could drastically depolarize DRG neurons, resulting in loss of excitability. We conclude that attenuation of pain sensation by I234T is caused by massively depolarized membrane potential of some DRG neurons which is partly due to enhanced overlap between activation and fast-inactivation, impairing their ability to fire. Our results demonstrate how a Na_v_1.7 mutation that produces channel gain-of-function can contribute to a dual clinical presentation that includes loss of pain sensation at the clinical level.

## Introduction

Sodium channel Na_v_1.7, encoded by the gene *SCN9A*, plays a major role in pain-signaling^1,2^. Point mutations of *SCN9A* that confer gain-of-function changes at the channel level have been linked to several pain disorders including inherited erythromelalgia (IEM)^3–6^ and paroxysmal extreme pain disorder (PEPD)^7–9^. Almost all of the Na_v_1.7 IEM mutations hyperpolarize activation, usually by 5–14 mV^2,3,10^, making it easier to activate the channel. When studied by current-clamp, these mutations produce a depolarization of RMP by 4–6 mV and increase the excitability of small-diameter DRG neurons which include nociceptors^11–13^. Lowered threshold and increased frequency of firing of DRG neurons underlie the pain experienced by patients with IEM who carry gain-of-function mutations in Na_v_1.7^14,15^.

Although the majority of IEM patients with Na_v_1.7 mutations manifest stereotypical symptoms of pain attacks in distal extremities triggered by mild warmth^16^, there have been cases with features of both IEM and PEPD^17,18^. Patients carrying the I234T mutation are unique in displaying a complex clinical phenotype, including a clinical gain-of-function (paroxysmal pain similar to the pain seen in IEM and PEPD)^18^ together with clinical loss-of-function features of impaired ability to sense pain, manifested by corneal anesthesia^19^, painless scratching, and painless pulling out of teeth^20^. The Na_v_1.7 I234T mutation massively hyperpolarizes activation by 18 mV, conferring a strong gain-of-function on the channel^18^. How a mutation of Na_v_1.7 that is gain-of-function at the channel level produces loss-of-function at the clinical level has remained enigmatic. Here we document additional forms of painless injuries in a patient carrying the I234T mutation, and demonstrate changes in RMP and excitability of some DRG neurons that contribute to the loss of pain sensibility in patients carrying this mutation.

## Materials and Methods

### Isolation and transfection of DRG neurons

All methods were performed in accordance with guidelines and regulations of Veterans Administration Connecticut Healthcare System and Yale University. Animal use was approved by the Veterans Administration Connecticut Institutional Animal Care and Use Committee. DRGs from female and male Sprague-Dawley rats were harvested and dissociated, and DRG neuron cultures were prepared as previously described^21^. DRG neurons from 4–6 week old rats were transfected with either WT or I234T hNa_v_1.7 channels together with EGFP in sister cultures; current-clamp experiments were performed 40–55 hrs afterwards. DRG neurons from 0–5 day postnatal rats transfected with either WT or I234T hNa_v_1.7 channels together with EGFP were used to assess the relationship between current threshold and membrane potential; current-clamp experiments were performed 20–32 hrs after transfection. Native DRG neurons from 0–5 day postnatal rats were used in dynamic-clamp experiments. We switched from adult to 0–5 day postnatal rats in a subset of our studies because of the failure to reproducibly maintain adequate clamp of adult DRG neurons for the hour-long extended protocol; cells are held at a series of membrane potentials when assessing the relationship between current threshold and membrane potential, as well as when injected with WT or I234T hNa_v_1.7 model that allows development of slow-inactivation.

### Current-clamp recordings

The electrophysiologist was blinded during data acquisition to whether DRG neurons were transfected with WT or I234T hNa_v_1.7 channels. The pipette solution contained (in mM): 140 KCl, 0.5 EGTA, 5 Hepes, and 3 Mg-ATP, 10 dextrose, pH 7.30 with KOH (adjusted to 310 mOsm with sucrose); the bath solution contained (in mM): 140 NaCl, 3 KCl, 2 MgCl_2_, 2 CaCl_2_, 10 Hepes, 10 dextrose, pH 7.30 with NaOH (adjusted to 320 mOsm with sucrose). Whole-cell configuration was obtained in voltage-clamp mode before proceeding to current-clamp. Recordings were obtained from small DRG neurons (<30 µm) with green fluorescence that displayed stable RMP (<10% variation) for 30 s and that expressed endogenous Na_v_1.8 currents (>1 nA evaluated at −50 mV)^22^. Input resistance was determined by the slope of a linear fit to hyperpolarizing responses to current steps from −5 to −40 pA in 5 pA increments. Current threshold was determined by the first action potential elicited by a series of depolarizing current injections (200 ms) in 5 pA increments. Action potential amplitude was measured from peak to RMP. Spontaneously-firing cells were excluded from analysis of action potential characteristics (i.e. current threshold, action potential amplitude, half-width and afterhyperpolarization potential).

### Dynamic-clamp recordings and kinetic models of WT and I234T hNa_v_1.7 channels

Dynamic clamp allowed us to replace 50% of the endogenous WT Na_v_1.7 current in DRG neurons with I234T current thus mimicking the heterozygous state. Membrane voltages were recorded in dynamic-clamp using a MultiClamp 700B amplifier (Molecular Devices) interfaced with CED Power1401 mk II DAI and Signal software (Cambridge Electronic Design, Cambridge, UK), digitized by Digidata 1440A DAC, and stored on hard disk using pCLAMP 10 software. Bath and pipette solutions were identical to those used for current-clamp recordings. We performed dynamic-clamp recording in DRG neurons using a model of Na_v_1.7 channel that was built based on experimentally determined Na_v_1.7 gating properties and the determination that Na_v_1.7 contributes on average 70% of the tetrodotoxin-sensitive (TTX-S) current in small DRG neurons; we based our estimate of the Na_v_1.7 contribution of 70% of the TTX-S current in small DRG neurons on our measurements of total TTX-S currents in Na_v_1.7 knock-out mice^23^.

We previously developed our model of Na_v_1.7 channel using Hodgkin-Huxley equations d*m*/d*t* = α_*m*_(1−*m*)−β_*m*_*m*; d*h*/d*t* = α_*h*_(1−*h*)−β_*h*_*h*, where *m* and *h* are activation and inactivation variables, and α(β) are forward (backward) rate constants, respectively^23^. WT and I234T steady-state parameters and kinetics were obtained based on electrophysiological recordings^18^ and converted into rate constants at respective voltages using the equations α = *m*_∞_/τ, β = (1−*m*_∞_)/τ. Reaction rate constants were fitted with Boltzmann equations of the form *y* = A2 + (A1−A2)/{1 + exp[(*V*−*V*_1/2_)/*k*]}, where *V* is membrane voltage, *V*_1/2_ is voltage when reaction rate is half-maximal, and *k* is slope coefficient. Fits were converted into steady-state variables and time constants according to *m*_∞_ = α/(α + β) and τ = 1/(α + β), respectively. The curves were overlayed on experimental data to provide feedback to the rate constants fitting step. This cycle was manually repeated until the best possible fit of experimental data was achieved.

Our previous model of Na_v_1.7 did not include slow-inactivation because we limited stimulation to 1 s trains where development of slow-inactivation is not appreciable. Here we added a slow-inactivation particle and used 30 s pulses to allow full development of slow-inactivation. Slow-inactivation time constants were obtained from recordings in HEK293 cells stably expressing WT or I234T hNa_v_1.7 (Supplementary Material).

We obtained the following rate constants for the WT Na_v_1.7 channel model:

α_*m*_ = 36.25 − 36.25/{1 + exp[(*V* − 19.00)/11.94]}, β_*m*_ = 27.25/{1 + exp[(*V* + 73.62)/13.64]};

α_*h*_ = 0.3156/{1 + exp[(*V* + 117.3)/11.11]}, β_*h*_ = 1.742 – 1.742/{1 + exp[(*V* + 8.942)/10.53]}.

α_*s*_ = 0.001886/{1 + exp[(*V* + 124.2)/8.336]}, β_*s*_ = 0.00033 – 0.00033/{1 + exp[(*V* + 32.67)/11.50]}.

And for I234T Na_v_1.7 model:

α_*m*_ = 41.54–41.54/{1 + exp[(*V*−16.80)/12.12]}, β_*m*_ = 16.75/{1 + exp[(*V* + 88.22)/9.843]};

α_*h*_ = 0.3156/{1 + exp[(*V* + 117.3)/11.11]}, β_*h*_ = 1.742–1.742/{1 + exp[(*V* + 8.942)/10.53]}.

α_*s*_ = 0.002439/{1 + exp[(*V* + 136.9)/10.76]}, β_*s*_ = 0.00034–0.00034/{1 + exp[(*V* + 57.60)/14.49]}.

Sodium current was described by *I*_Na_ = *g*_max_ × *m*^3^ × *h* × *s* × (*V*_m_ − *E*_Na_), where *V*_m_ is membrane voltage potential and *E*_Na_ = 65 mV is sodium reversal potential. Currents evoked by different voltage protocols were calculated in 10-μs precision using a custom program written in Origin 8.5 LabTalk.

### Statistical analysis

Data analysis used Fitmaster (HEKA Elektronik) and Origin (Microcal Software, Northampton, MA). Unless otherwise noted, statistical significance was determined using an independent *t* test. Percentage of non-firing cells was compared using *z* test. Results are presented as mean ± standard error of means (SEMs); error bars represent SEMs. Statistical significance is shown by asterisks *indicating *p* < 0.05, **indicating *p* < 0.01 and ***indicating *p* < 0.001.

## Results

### I234T phenotype includes loss of pain sensibility as well as episodic pain

The first report of a child carrying the Na_v_1.7 I234T mutation included clinical features of both IEM and PEPD^18^. The mutation was heterozygous (carried on one *SCN9A* allele). Episodes of severe pain, in some cases provoked by minor falls, began at age 15–16 months. Tactile stimulation around the sacrum provoked attacks, similar to PEPD, although bowel movement was not a trigger and there was no flushing. The patient was noted to sweat but was intolerant of heat; the limbs would flush or become reddish-purple in response to warmth. The patient subsequently developed nearly-daily paroxysmal “lightning” pain, occasional facial pain, and prolonged episodes of burning pain of the feet and hands triggered by heat or fatigue. Multiple trials of pharmacotherapy did not provide relief.

In addition to these painful symptoms, the patient’s clinical phenotype includes painless injuries. The patient displayed severe joint hypermobility and altered gait. Biting and rubbing the skin on the hands and feet resulted in skin breakdown. Repeated scratching resulted in scarring around the eye, and collapse of the nasal septum. After the initial reporting of this subject^18^, the patient developed a spiral fracture of the left femur at age 10 through over-stretching, but did not complain of pain and walked on the swollen leg for a full day before X-ray revealed a fracture. The patient does not appear to feel pain to venipuncture or introduction of venous catheters. Intragluteal injections are not felt as painful, although they sometimes produce a sensation of pressure. In contrast, injections in the thigh were felt as burning pain. At 12 years of age, the patient developed a large pelvic abscess that required drainage. This presented with urinary retention, abdominal distension, and high inflammatory markers (white cell count and C-reactive protein) without a significant painful response. We evaluated the effect on DRG neurons of the expression of the I234T channel in order to determine if we could detect a cellular correlate to the complex of painful symptoms and painless injuries manifested in patients carrying the I234T mutation.

### Expression of I234T silences some DRG neurons

**Figure 1:**
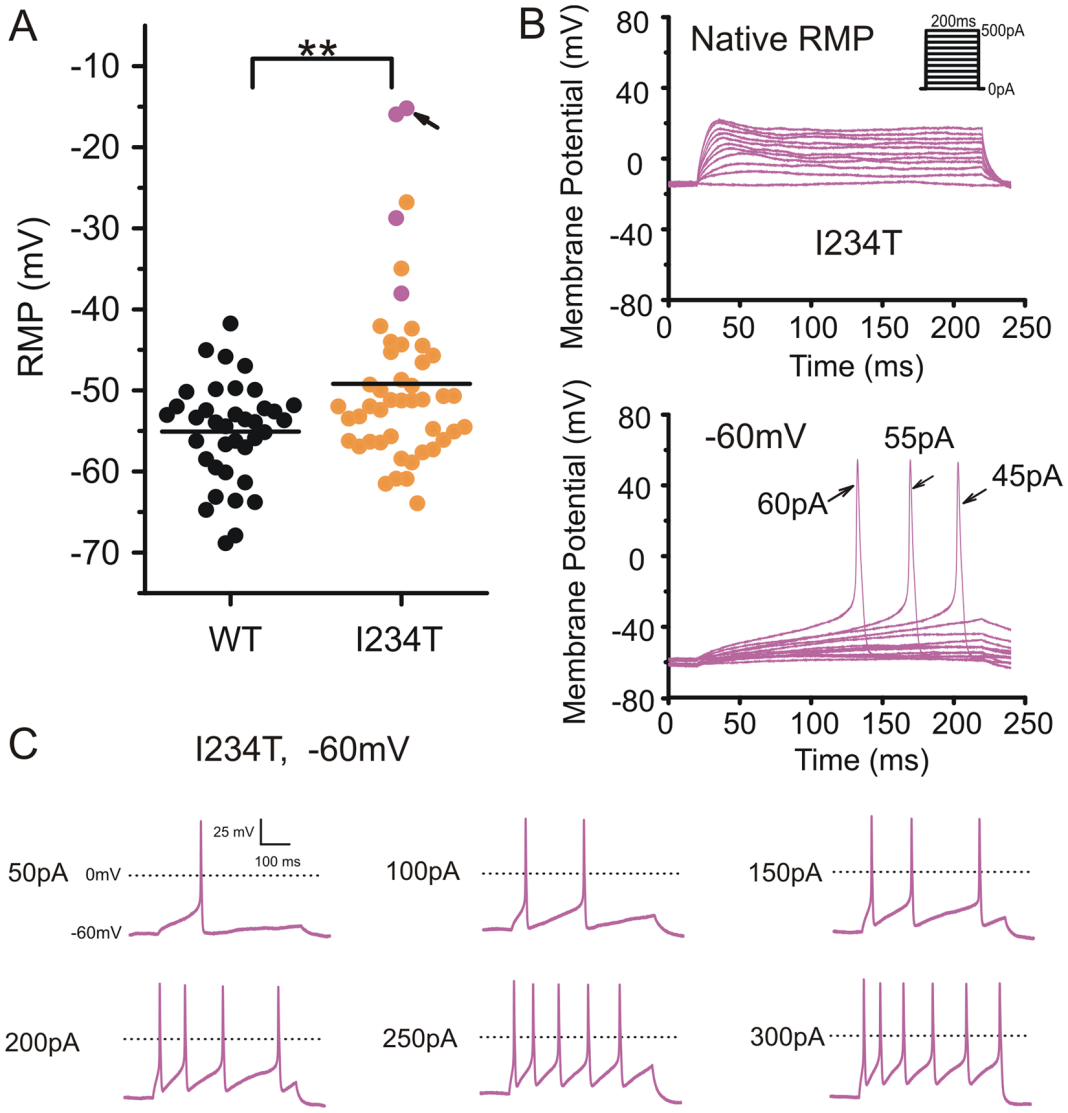
Expression of I234T leads to massive depolarization of RMP and inexcitability in a small number of DRG neurons. (**A**) Scatter-plot of native resting potentials in neurons expressing either WT or I234T hNa_v_1.7 channels. Filled circles in magenta indicate cells that do not fire any all-or-none action potentials in response to external stimuli at their native resting potentials but regain excitability when held at −60 mV. **Indicates statistical significance (*p* < 0.01). (**B**) A small DRG neuron with a native resting potential of −15.2 mV (arrow in **A**) does not produce action potentials in response to 200 ms current injections from 0 to 500 pA in 50 pA increments (upper). When held at −60 mV, this cell generates action potentials when stimuli exceed 45 pA (lower). Arrows with numbers indicate stimuli used to elicit the labeled response. (**C**) The same neuron in (**B**) which does not fire action potentials at its native resting potential of −15.2 mV, when clamped at −60 mV, fires repetitively in response to 500 ms current injections that are multifold of its current threshold (i.e. 45 pA). Numbers to the left of action potential traces indicate the stimuli.

We used current-clamp recording to assess the effect of expression of I234T mutant channels on DRG neurons. DRG neurons expressing I234T exhibited a wider range of RMP (−63.9 mV to −15.2 mV, *n* = 46), compared to cells expressing WT (−68.9 mV to −41.7 mV, *n* = 37). On average, cells expressing I234T showed a significantly more depolarized RMP compared to WT (WT: −55.1 ± 1.0 mV, *n* = 37; I234T: −49.2 ± 1.6 mV, *n* = 46; *p* = 0.00384) (Table 1), and more cells expressing I234T displayed strongly depolarized membrane potentials (Fig. 1A).

**Table 1:** Current-clamp characteristics of DRG neurons expressing WT or I234T Na_v_1.7 measured at native resting potentials.

hNa_v_1.7	Resting membrane potential	Non-firing cells	Input resistance	Current threshold	AP amplitude	Half-width	Afterhyperpolarization potential
	(mV)	(%)	(MΩ)	(pA)	(mV)	(ms)	(mV)
WT	−55.1 ± 1.0 (*n* = 37)	0/37, 0%	513 ± 47 (*n* = 37)	263 ± 28 (*n* = 37)	106 ± 2.2 (*n* = 37)	4.97 ± 0.42 (*n* = 37)	−64.5 ± 1.3 (*n* = 37)
I234T	−49.2 ± 1.6** (*n* = 46)	4/46, 8.7%**	430 ± 33 (*n* = 46)	194 ± 22 (*n* = 42)	105 ± 2.4 (*n* = 42)	4.83 ± 0.37 (*n* = 42)	−60.9 ± 1.3 (*n* = 42)

**Indicates *p* < 0.01.

Although no cell expressing WT displayed a RMP more positive than −40 mV, 13% of cells expressing I234T (6 out of 46 cells) had RMPs more positive than −40 mV. Notably, 4 out of 46 cells expressing I234T (8.7%) (Table 1), with RMPs of −15.2 mV, −16.0 mV, −28.8 mV and −38.0 mV (magenta, Fig. 1A), remained silent in response to external stimuli. Input resistances of these cells were 116 MΩ, 368 MΩ, 220 MΩ and 225 MΩ, respectively. We predicted that depolarized RMPs contributed to inexcitability of these DRG neurons. To test this hypothesis, we imposed a relatively normal membrane potential of −60 mV in these cells by injecting hyperpolarizing current, and observed that all four cells regained excitability. For these four neurons, the current thresholds for action potential generation from a holding potential of −60 mV were 45 pA, 90 pA, 225 pA and 415 pA, respectively. An example is provided by the cell with a native resting potential of −15.2 mV (arrow, Fig. 1A), which failed to produce an action potential with stimuli as large as 500 pA applied from this native resting potential (Fig. 1B, upper), but fired an action potential with a current threshold of 45 pA (Fig. 1B, lower) when held at −60 mV, and fired repetitively in response to graded suprathreshold stimuli (Fig. 1C).

### Expression of I234T reduces threshold and increases firing frequency in DRG neurons excitable at native resting potential

**Figure 2:**
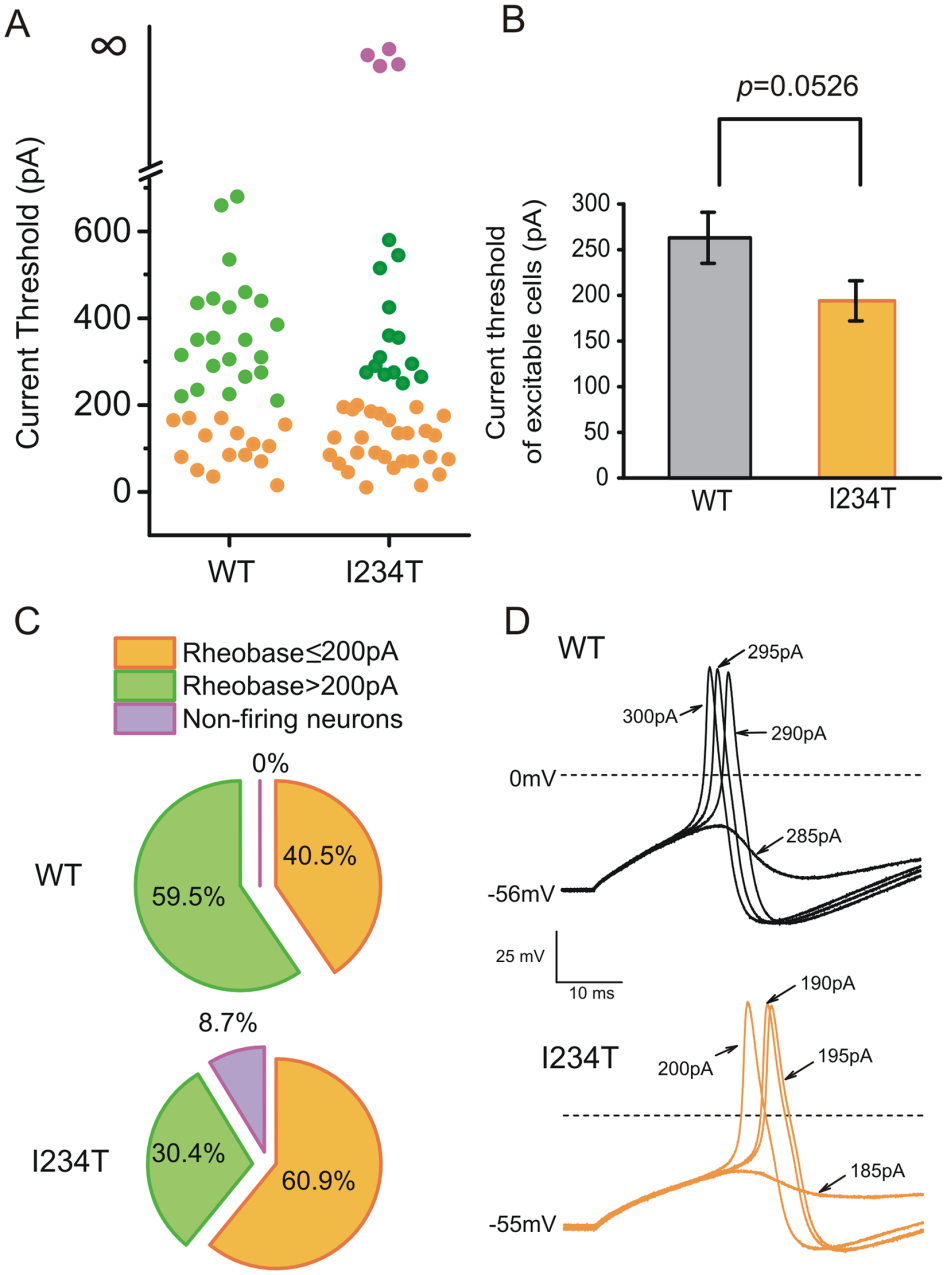
Expression of I234T tends to lower current threshold in DRG neurons that are excitable at native resting potential. (**A**) Scatter-plot of current threshold in neurons expressing either WT or I234T. Filled circles in magenta represent silent cells that did not fire in response to external stimuli from their native resting potentials but regain excitability when held at −60 mV. Their current thresholds at native potentials were considered infinite. (**B**) In neurons that were excitable at native resting potentials, I234T tended to reduce average current threshold, compared to WT. (**C**) Populations of cells with current thresholds ≤ 200 pA (orange), > 200 pA (green) and silent (magenta) at their native resting potentials. (**D**) Representative responses of neurons expressing WT or I234T to 200 ms current injections in 5 pA increments. The cell expressing WT had an RMP of −56 mV and a current threshold of 290 pA (upper). The neuron expressing I234T had an RMP of −55 mV and generated an action potential at a threshold of 190 pA (lower).

I234T did not affect average input resistance, amplitude, half-width or afterhyperpolarization of action potentials (Table 1). Excluding the four inexcitable neurons (magenta, Fig. 2A), there was a tendency toward reduction in current threshold by 26% for cells expressing I234T compared to WT (Fig. 2B). A significantly smaller proportion of cells expressing I234T (30.4%) displayed high current thresholds (>200 pA) compared to cells expressing WT (59.5%, *p* = 0.0078) (Fig. 2C). Representative action potential traces from small DRG neurons expressing WT or I234T are shown in Fig. 2D. The cell expressing WT channels had an RMP of −56 mV and generated an action potential when the current injection reached 290 pA (upper panel, Fig. 2D). The native resting potential for the neuron expressing I234T was −55 mV and it generated an action potential at a threshold of 190 pA (lower panel, Fig. 2D).

**Figure 3:**
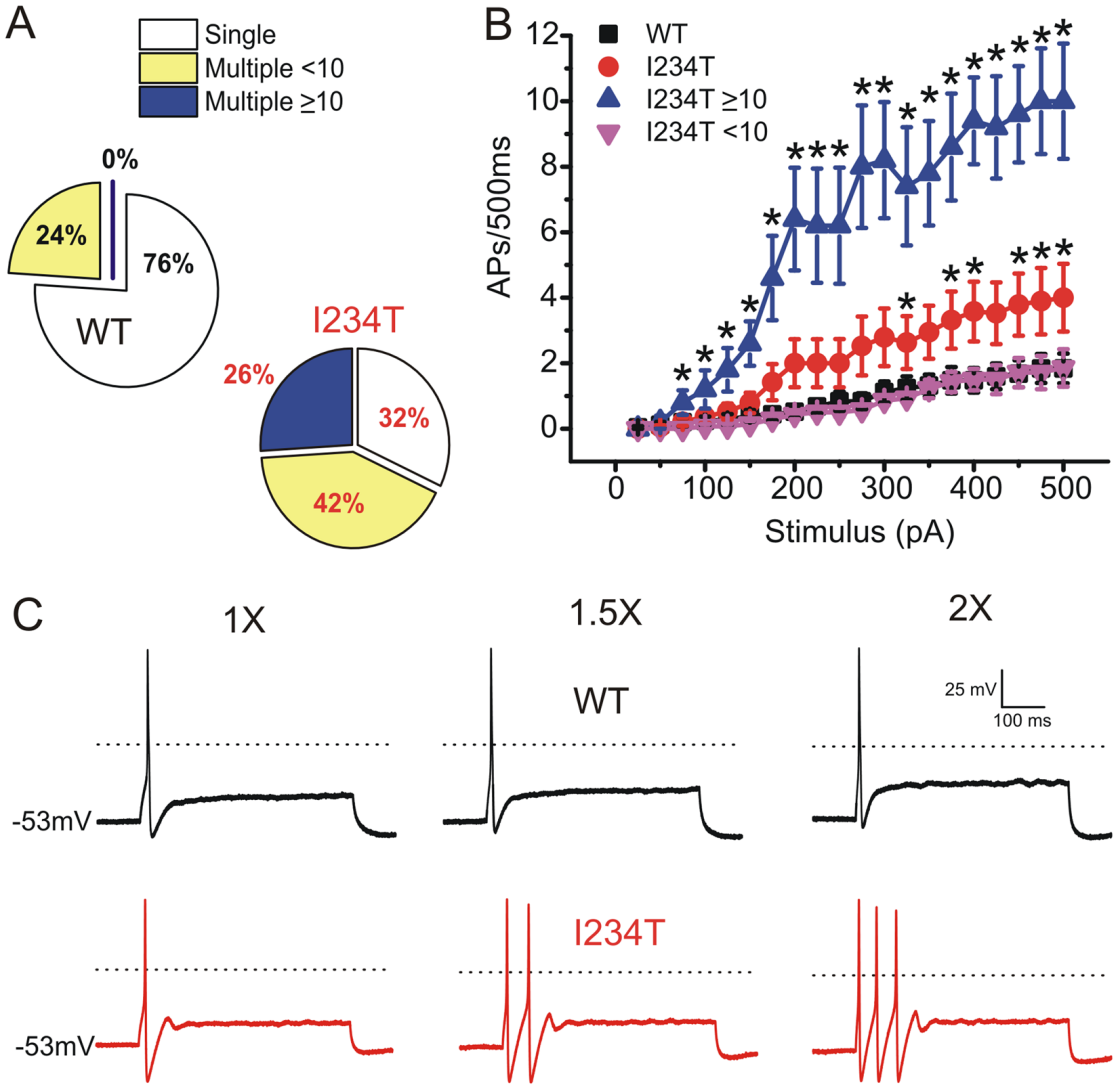
Expression of I234T produces hyperexcitability in DRG neurons that are excitable at native resting potential. (**A**) Populations of cells expressing either WT or I234T maximally fired single action potential (white), < 10 (yellow) and ≥ 10 action potentials (blue) in response to graded 500 ms current stimuli of 25 to 500 pA in 25 pA increments. (**B**) Average firing frequencies in cells expressing WT (black) or I234T (red) in response to 500 ms graded inputs from 25 to 500 pA in 25 pA increments. Cells expressing I234T were divided into high- and low-firing groups, which elicited maximally ≥10 (blue) and <10 (magenta) action potentials over 500 ms, respectively. (**C**) Responses of a DRG neuron expressing WT (upper) or I234T (lower) to 500 ms depolarizing current steps that are 1-, 1.5- and 2-fold current threshold, respectively.

In response to graded suprathreshold stimuli, the majority of DRG neurons expressing WT (76%, 19 out of 25 cells) fired single action potentials during 500 ms (Fig. 3A). In contrast, the majority of DRG neurons expressing I234T fired repetitively (68%, 13 out of 19 cells). Among these repetitive-firing neurons, 26% of them (Fig. 3A,B, blue) fired at a much higher frequency (more than ten action potentials per 500 ms) than cells expressing WT. On average, DRG neurons expressing I234T (red) fired more action potentials than WT (black) (Fig. 3B). Even for cells that have a similar resting potential, the expression of I234T caused increased firing compared to the expression of WT channels (Fig. 3C).

### Action potential current threshold is related to membrane potential

As shown above, 8.7% of DRG neurons expressing I234T were inexcitable at their massively depolarized native resting potentials; however, these cells regained excitability when held at −60 mV. To examine the effect of RMP on neuronal excitability in single cells with a range of native RMPs, precisely calibrated inward currents were injected into each cell to clamp the membrane potential at a series of voltages starting from −60 mV in 5 mV increments. In a different set of cells than that reported above, I234T significantly depolarized RMP in DRG neurons compared to WT (WT: −53.6 ± 1.4 mV, *n* = 16; I234T: −41.8 ± 4.0 mV, *n* = 14; *p* = 0.00668). As expected, 3 out of 14 DRG neurons expressing I234T displayed markedly depolarized native RMPs of −10.1 mV, −20.7 mV and −21.6 mV, respectively, and none of these cells fired action potentials from their native RMPs. All three cells generated action potentials in response to current injections from a holding potential of −60 mV, with current thresholds of 110 pA, 190 pA and 55 pA, respectively.

**Figure 4:**
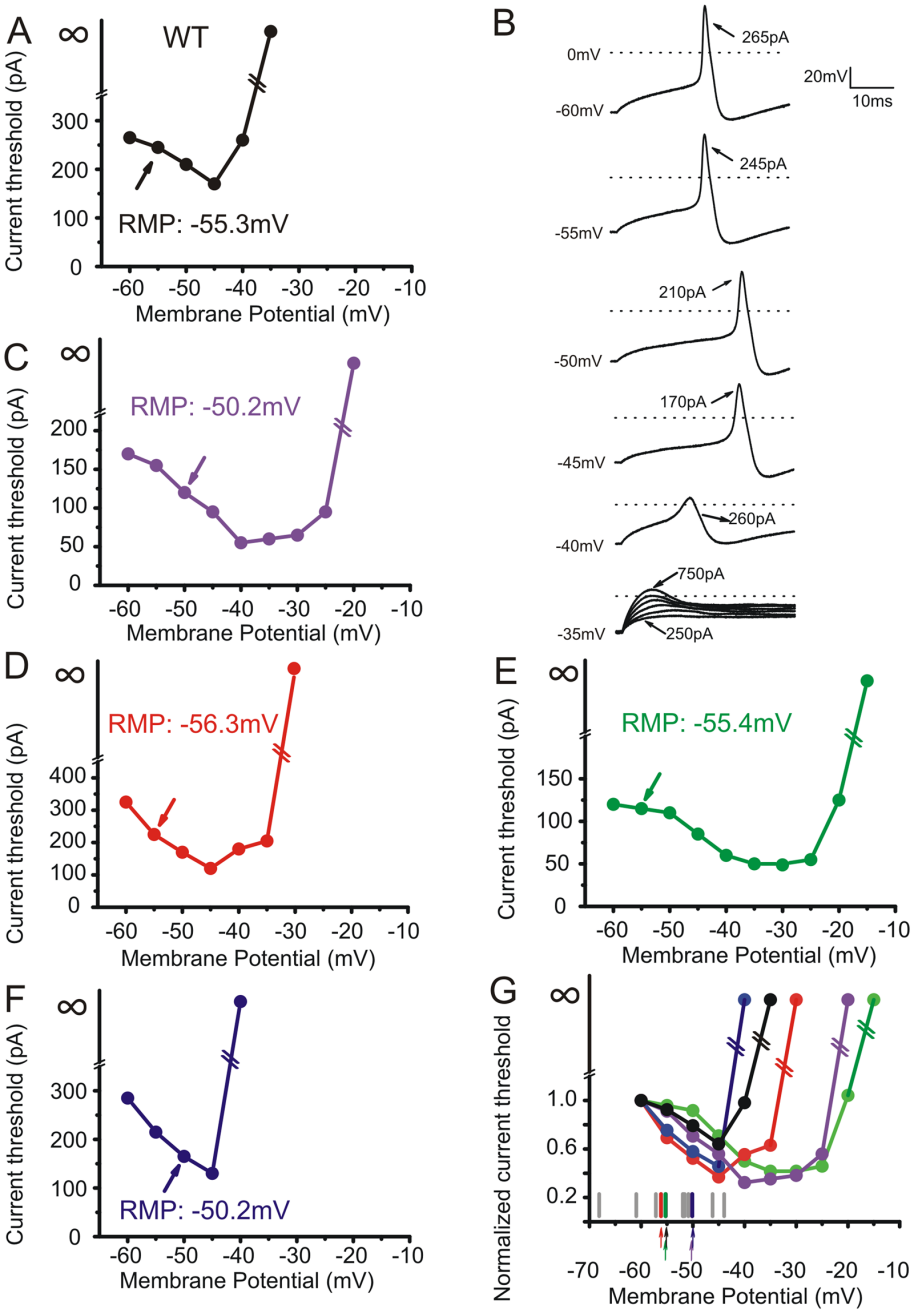
Depolarization of RMP causes biphasic changes in current threshold in DRG neurons expressing WT hNa_v_1.7 channels. DRG neurons expressing WT were held at an initial membrane potential of −60 mV and depolarized in 5 mV increments until cells become inexcitable. The relationship between current threshold and membrane potential is U-shaped in five individual cells (**A**,**C**–**F**). Note that, for each cell, the bottom of the curve is minimal current threshold. (**B**) Representative traces from the cell illustrated in (**A**) at holding potentials indicated to the left. (**G**) Superimposition of curves from five cells expressing WT, with current threshold normalized to the value at holding potential of −60 mV. The distribution of RMPs in a total of 16 cells is illustrated under the curves. Arrows with matching colors indicate RMPs from representative cells shown in (**A**,**C**–**F**).

Figure 4 plots current threshold for action potential generation in neurons expressing WT as membrane potentials were depolarized from an initial holding potential of −60 mV. Current threshold decreased to a minimum with progressively increased depolarization until a critical point at which further depolarization produced an increase in current threshold. Further depolarization silenced these neurons even in response to stimuli as large as hundreds of pA. The U-shape relationship between membrane potential and current threshold is illustrated for five neurons expressing WT (Fig. 4A,C–F). Representative action potential traces from the neuron illustrated in Fig. 4A at various membrane potentials are shown in Fig. 4B. This neuron had a native resting membrane potential of −55.3 mV and its current threshold at this native resting potential was 245 pA (Fig. 4A, arrow). When membrane potential was depolarized to −35 mV no action potentials could be evoked even when the injected current was increased to 750 pA (Fig. 4B), and the current threshold was shown as infinite in Fig. 4A. Current thresholds for all five neurons expressing WT at various holding potentials were normalized to the current threshold at −60 mV for comparison, and plotted against holding membrane potential in Fig. 4G. The RMPs from all 16 cells expressing WT are shown under the curves in Fig. 4G, with colored arrows indicating RMPs from the representative curves. Although there is a wide range of the minima where threshold for each cell was the lowest, each cell expressing WT tended to display a native RMP more hyperpolarized than the critical minimum.

**Figure 5:**
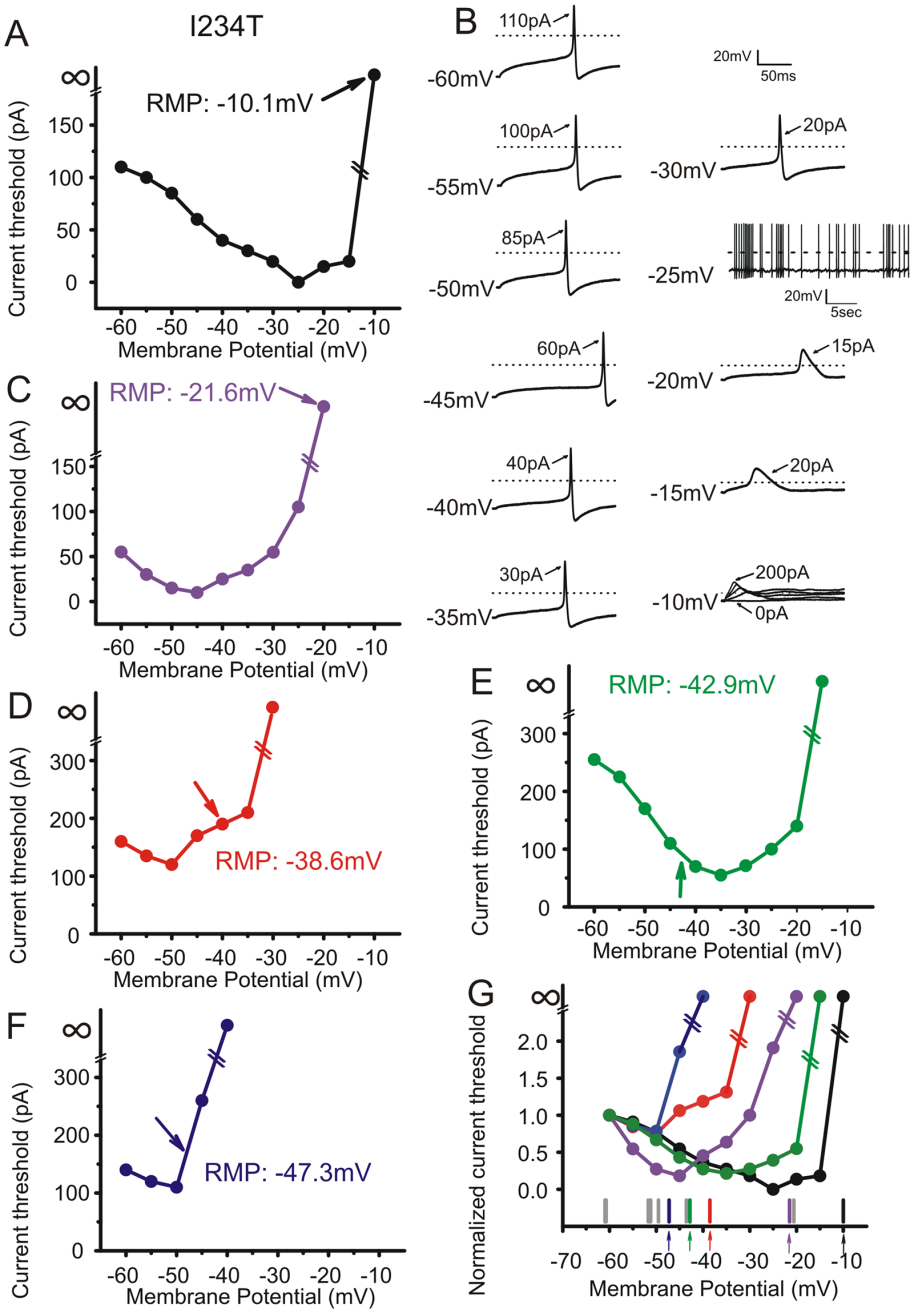
Depolarization of RMP causes biphasic changes in current threshold in DRG neurons expressing I234T mutant channels. The curve depicting the relationship between current threshold and membrane potential is U-shaped in five individual cells expressing I234T (**A**,**C**–**F**). (**B**) Representative traces recorded from the cell illustrated in A at holding potentials indicated to the left. (**G**) Superimposition of curves from five cells expressing I234T with current threshold normalized to the value at a holding potential of −60 mV. The distribution of RMPs in a total of 14 cells expressing I234T is illustrated under the curves. Arrows with matching colors indicate RMPs from representative cells shown in (**A**,**C**–**F**).

Figure 5 shows similar plots for DRG neurons expressing I234T, and demonstrates that RMPs of some cells expressing I234T mutant channels are more depolarized than the critical minimum (determined by holding cells at −60 mV). Current threshold-membrane potential curves from five DRG neurons expressing I234T are shown (Fig. 5A,C–F). Figure 5B displays representative action potential traces from the neuron illustrated in Fig. 5A. The native RMP for this neuron was massively depolarized (−10.1 mV). The cell failed to fire action potentials from this depolarized RMP. However, it generated overshooting action potentials when its membrane potential was held at more negative potentials. For example, the cell fired action potentials in response to current injection of 110 pA when it was held at −60 mV, and fired spontaneously when held at −25 mV, a membrane potential close to the voltage threshold for action potentials. Further depolarization to −10 mV silenced the neuron, as was observed at this cell’s native resting potential (Fig. 5B). In contrast to cells expressing WT showing their native RMPs more hyperpolarized than the critical minimum (Fig. 4G), cells expressing I234T tended to display a native RMP more depolarized than the critical minimum, as indicated by arrows in Fig. 5G.

### Dynamic-clamp confirms that massive depolarization of RMP can reduce excitability in DRG neurons with physiological levels of I234T

The results above indicate that expression of I234T massively depolarizes RMP in a small number of DRG neurons and suggest that this depolarization silences these neurons. Since these results were obtained in transfected cells where it is not possible to control sodium channel expression level, we performed dynamic-clamp experiments where we could precisely titrate the levels of WT and I234T currents. In order to build the models of WT and I234T Na_v_1.7 channels, we determined the channel repriming rates from fast- and slow-inactivation in HEK293 cells stably expressing either WT or I234T (Figure S1). Our models of WT and I234T Na_v_1.7 channels include all three m, h and s gates (Fig. S2), and accurately reproduced steady-state properties of the channel (Table S1).

**Figure 6:**
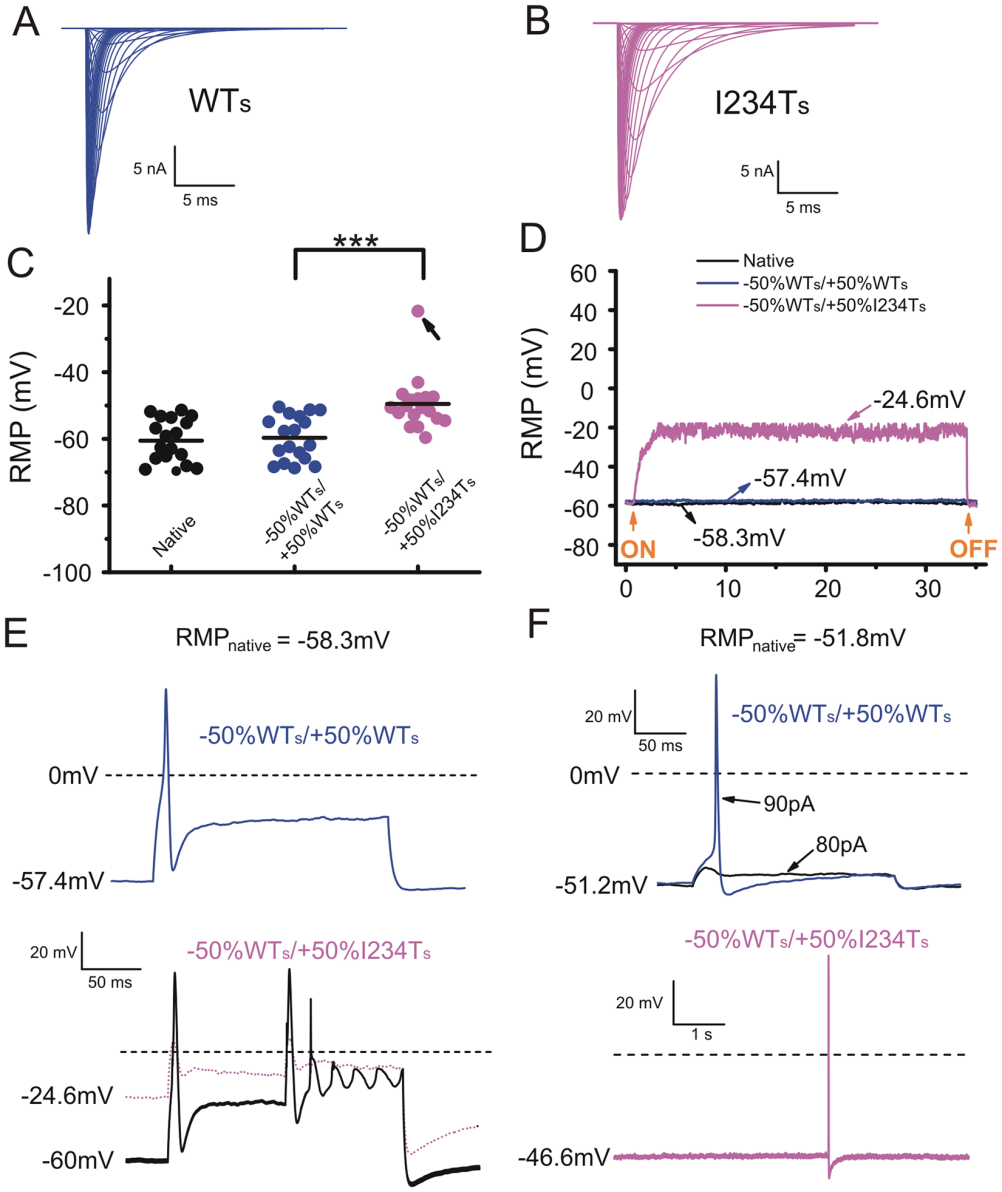
Dynamic-clamp injection of I234T conductance can lead to massive depolarization of RMP in native DRG neurons. Simulated wild-type (WT_s_, **A**) and mutant (I234T_s,_ **B**) hNa_v_1.7 currents. (**C**) Distributions of RMPs in the following three conditions: no model implemented (native Na_v_1.7 current), replacing 50% endogenous WT Na_v_1.7 conductance with an equal amount of WT_s_ current (−50% WT_s_/ + 50% WT_s_), and replacing 50% endogenous WT Na_v_1.7 conductance with an equal amount of I234T_s_ (−50% WT_s_/ + 50% I234T_s_). (**D**) The RMP of a cell endowed with I234T_s_ conductance was depolarized to −24.6 mV (arrow in **C**). RMP was recorded continuously for 30 s after injecting either 50% WT_s_ or 50% I234T_s_ Na_v_1.7 conductance. (**E**) (upper) In the cell as in (**D**), an action potential was evoked in response to 200 ms current injection of 500 pA when endowed with WT_s_ conductance. (lower) This cell when endowed with I234T_s_ conductance, rested at −24.6 mV and failed to produce an action potential; when held at −60 mV in the presence of I234T_s_ conductance, it regained excitability. (**F**) In another cell that rested at −51.8 mV, an action potential was generated when current stimulus was increased from 80 pA to 90 pA (upper). When I234T_s_ was introduced, RMP was depolarized to −46.6 mV and the cell fired spontaneously (lower).

This modeled conductance permitted us to compare, in the same cell, the effects of addition of a physiological level of I234T versus WT Na_v_1.7 conductance (Fig. 6A,B). Prior to turning on the dynamic clamp, the averaged RMP for the native DRG neurons was −60.5 ± 1.5 mV (*n* = 18). The endogenous Na_v_1.7 conductance was estimated based on an assumption that Na_v_1.7 contributes on average 70% of the TTX-S current in small DRG neurons, which was determined by comparing TTX-S currents in small DRG neurons in wild-type and Na_v_1.7-null mice; this conductance was calculated to be 250 nS^23^. We first removed 50% (125 nS) of the endogenous conductance and replaced with an equal value (125 nS) using our modeled WT Na_v_1.7 to mimic a homozygous WT neuron. This maneuver, as expected, barely changed RMP (RMP = −59.7 ± 1.6 mV, *n* = 18, *p* = 0.715), increasing confidence that implementation of dynamic clamp protocols did not significantly alter the membrane properties of these neurons. Replacement of the endogenous 125 nS with modeled I234T conductance was used to mimic the heterozygous state in patients carrying this mutation. This maneuver resulted in a significant depolarization of averaged RMP to −49.7 ± 1.8 mV (*n* = 18, *p* = 1.46E-4; Fig. 6C).

In a native small DRG neuron resting at −58.3 mV (Fig. 6C, arrow), replacement of 50% endogenous WT with 50% I234T conductance massively depolarized RMP to −24.6 mV (Fig. 6D), a 33.7 mV shift, and silenced the cell to 200 ms/500 pA stimulus, but action potential electrogenesis was rescued when the cell was held at −60 mV (Fig. 6E, lower). In contrast, replacement with 50% WT conductance slightly depolarized RMP by 0.9 mV and, in response to 200 ms/500 pA current injection, the cell fired a typical action potential (Fig. 6E, upper). This observation recapitulated our findings that overexpression of I234T channel caused a small number of DRG neurons to drastically depolarize their RMPs, thereby suppressing their excitability.

**Figure 7:**
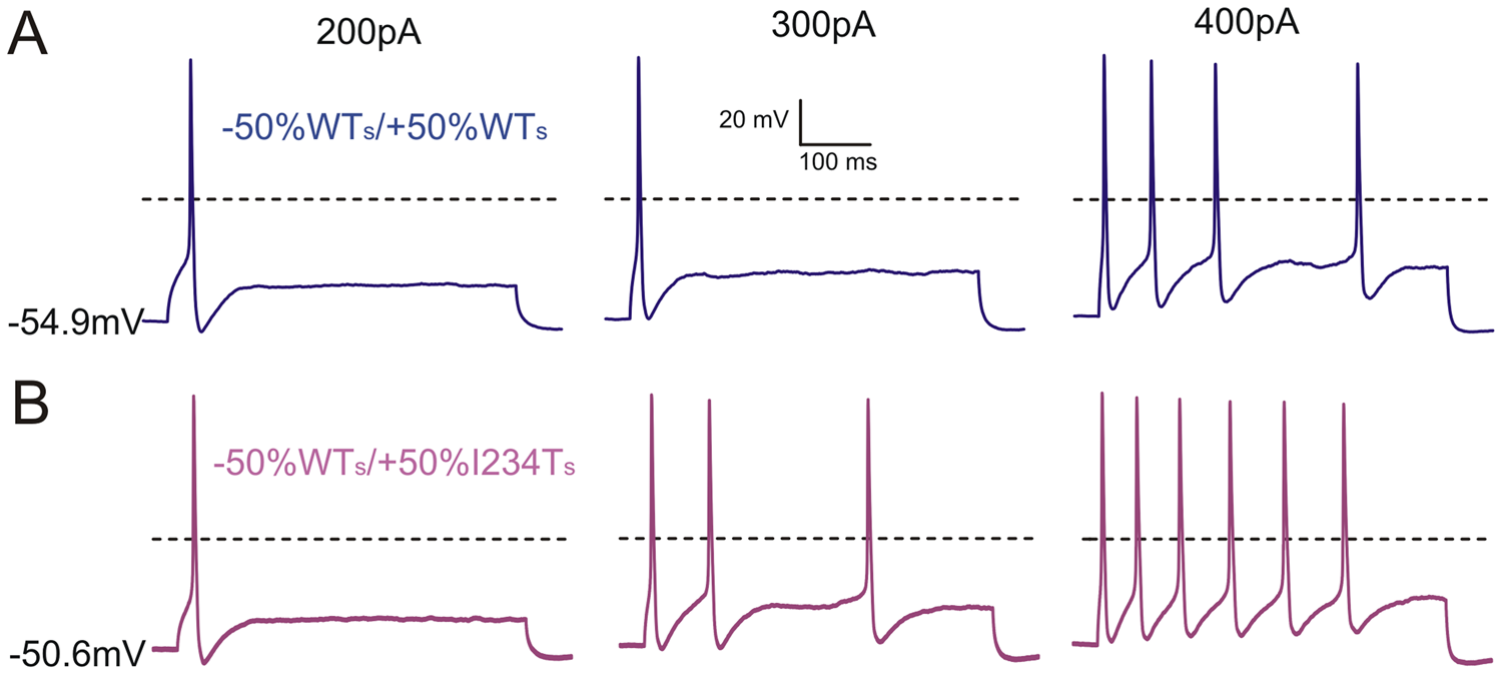
Injecting simulated I234T conductance using dynamic-clamp results in hyperexcitability in some native DRG neurons. The cell shown here initially had an RMP of −56.8 mV. Replacing 50% of WT Na_v_1.7 conductance with WT_s_ (**A**) depolarized RMP by 1.9 mV while introducing 50% I234T_s_ (**B**) depolarized RMP by 6.2 mV. In response to 500 ms current stimuli of 200 pA, a single action potential was evoked in the presence of either WT_s_ or I234T_s_ conductance. Introduction of I234T_s_ produced hyperexcitability in the same cell, manifested as increased numbers of action potentials in response to 300 pA and 400 pA current stimuli.

In contrast to this massive depolarization of RMP that leads to neuronal hypoexcitability, 5–6 mV depolarizations in RMP in other DRG neurons produced hyperexcitability. For example, in a cell with native RMP of −51.8 mV, the current threshold for action potential generation was 90 pA when 50% of the endogenous conductance was replaced by 50% modeled WT Na_v_1.7 (Fig. 6F, upper). The RMP in this cell was depolarized by 5.2 mV by endowing the cell with 50% I234T conductance, which led to spontaneous firing (Fig. 6F, lower). In another cell which rested at −56.8 mV, electronic introduction of WT or I234T Na_v_1.7 conductance depolarized its RMP to −54.9 mV and −50.6 mV, respectively (Fig. 7A,B). As compared to WT, the 6 mV RMP depolarization associated with 50% I234T conductance was accompanied by higher frequencies of evoked firing in response to graded suprathreshold stimuli (Fig. 7A,B).

## Discussion

While most gain-of-function mutations that enhance activation of Na_v_1.7 produce a relatively stereotyped syndrome of IEM characterized by intense pain^2,16^, some mutations are associated with atypical clinical syndromes. The I234T Na_v_1.7 mutation produces an unusually large hyperpolarization of activation, i.e. a strong enhancement of activation which is gain-of-function at the channel level^18^. The two patients carrying this mutation displayed a complex picture of the painful symptoms as reported by patients with IEM and PEPD, together with a painless fracture, absence of pain during venipuncture, and absence of pain in response to intragluteal injections, and corneal anesthesia suggesting pain insensitivity within territories innervated by trigeminal ganglion neurons^18–20^. The patient reported in this study did not complain of pain in association with a pelvic abscess. The coexistence of somatic painful symptoms together with the absence of pain in association with bone fractures, venipunctures, and corneal injury in two unrelated patients carrying the I234T mutation suggests a common mechanistic link that is related to this mutation rather than another genetic or epigenetic factor(s).

We demonstrate here that the I234T mutant channel massively depolarizes resting potential in ~10% of transfected DRG neurons from average RMP of −50 to −55 mV to voltages more positive than −40 mV, in some cases to nearly −15 mV. This is in contrast to most IEM mutations, which depolarize RMP of DRG neurons by 5–6 mV, a shift which, in itself, produces DRG neuron hyperexcitability^11–13^. Our voltage-clamp assessment at 33 °C revealed, in addition to massive overlay of activation and steady-state fast-inactivation, an approximately 60% increase in persistent current (Fig. S3H), which would be expected to contribute to depolarization of RMP. Previous dynamic-clamp recordings in cells using conductances modeled after other Na_v_1.7 IEM mutations indicate that increased overlap between activation and fast-inactivation, due to hyperpolarizing activation, produces a sustained “window current” that acts, together with persistent current, to depolarize RMP^23^. The unusually large hyperpolarization of activation of I234T would be expected to produce a markedly enhanced window current, predicting a large depolarization of RMP in DRG neurons expressing I234T. The mechanistic basis for the larger variability in RMP in cells expressing I234T mutant channels, and the factors that differentiate massively depolarized neurons from less severely depolarized cells, are not clear, although we have previously shown that the magnitude of the late current and the starting RMP of the neuron impact the magnitude of RMP depolarization caused by the presence of a mutant Na_v_1.7 conductance^24^. The late current, however, was not increased at room temperature in I234T mutant channels^18^. Irrespective of this, our dynamic-clamp data confirmed a much larger depolarization in average RMP, including a massive depolarization in RMP to approximately −25 mV in one cell after implementing 50% I234T conductance, a condition that mimics the heterozygous condition in patients. This large depolarization would be expected to inactivate all sodium channels, including Na_v_1.8, in the cell in question.

Consistent with a failure of action potential electrogenesis in DRG neurons expressing I234T mutant channels in which RMP was massively depolarized, the amplitude of action potentials recorded from non-transfected small DRG neurons is dependent on holding potential, and this voltage-dependence is best fit with two Boltzmann equations^25^. The more hyperpolarized inactivation V_1/2_ was similar to the V_1/2_ for TTX-S sodium channels and was eliminated by 300 nM TTX, indicating a contribution of TTX-S currents. The V_1/2_ for the more depolarized component was similar to the V_1/2_ of fast-inactivation for the TTX-resistant Na_v_1.8 sodium channel. When Na_v_1.8^(−/−)^ DRG neurons were transfected with Na_v_1.8, the voltage-dependence of action potential amplitude was rescued, and recapitulated the voltage-dependence observed in neurons from WT animals^25^. These observations indicate that Na_v_1.8 can support action potential electrogenesis in DRG neurons when these cells are moderately depolarized, and show that, when these cells are depolarized to a voltage more positive than −25 mV where Na_v_1.8 is not operable, action potential electrogenesis is compromised.

The episodic burning pain in our patient is exacerbated by heat. Other reports have studied Na_v_1.7 mutations at more physiologically relevant temperatures by voltage-clamp^26,27^ and current-clamp measurements in DRG neurons and iPSC-derived sensory neurons^15,28^. To understand the mechanism underlying the heat-aggravated pain sensation in the patient, we examined the biophysical properties of I234T mutant channels at a physiologically relevant temperature (Supplementary Material). At 33 °C, physiological skin temperature, we observed gain-of-function attributes of I234T channels that were similar to those at room temperature^18^ (Table S2), including a massive hyperpolarizing shift in activation (−14 mV, Fig. S3A,B), enhancement of ramp currents by approximately 5 fold (Fig. S3C,D), and deceleration of deactivation kinetics (Fig. S3E). The voltage-dependence of steady-state fast-inactivation remained unaffected by the I234T mutation at this higher temperature (Fig. S3F). The loss-of-function attribute of I234T, i.e. the hyperpolarizing shift in voltage-dependence of slow-inactivation, however, was reduced from a shift of 21 mV at room temperature^18^ to 10 mV at 33 °C (Fig. S3G). Moreover, the late (persistent) current was significantly increased in I234T mutant channel at 33 °C (Table S2, Fig. S3H,I,J). Although the increase in late current was not observed at room temperature in our previous study^18^, the enhanced late current at 33 °C might constitute another gain-of-function attribute that contributes to the clinical phenotype *in vivo*. Diminished loss-of-function attribute, together with enhanced gain-of-function features, would be expected to enhance the proexcitatory contribution of I234T mutant channel, and this may account for the exacerbation of pain by warmth in this patient.

The loss of pain sensation in the patient reported here was not total because the patient did not feel pain with intragluteal injections, but felt burning pain in response to injections in the thigh. While we do not fully understand this complex clinical phenotype in which some forms of painful sensation are lost while others are retained, it is notable that it is paralleled by a large range of native resting potentials, accompanied by a spectrum of excitability in DRG neurons expressing I234T, with some cells displaying massively depolarized native RMP and loss of excitability, and others displaying hyperexcitability at their less depolarized resting potentials. Other cases of a dual pain phenotype with combined painful and painless symptoms in patients with gain-of-function mutations in sodium channel Na_v_1.9 have been recently described^29–33^. Like the I234T Na_v_1.7 mutation, the mutant Na_v_1.9 channels from these patients with painless symptoms produce massive depolarization of RMP and loss of excitability in DRG neurons^33^. Thus, the concept of gain-of-function mutations leading to loss of pain sensibility via massive depolarization of RMP in DRG neurons appears to be generalizable beyond the Na_v_1.7 channel. Further studies will be needed to characterize the multiple active and passive membrane parameters that contribute to the range of RMPs that we observed in DRG neurons, and to the larger depolarization of RMP in some neurons upon expressing I234T. Irrespective of this latter consideration, our findings on DRG neurons expressing the Na_v_1.7 I234T mutation indicate that loss of pain sensibility in patients carrying a Na_v_1.7 mutation which hyperpolarizes activation to an unusually great degree, is due to massive depolarization of the RMPs of some DRG neurons, impairing their ability to fire.

## Electronic supplementary material

Supplementary Material
