# Supplementary material for: Atypical changes in DRG neuron excitability and complex pain phenotype associated with a Nav1.7 mutation that massively hyperpolarizes activation
Source: Sci Rep. 2018 Jan 29;8:1811. doi: 10.1038/s41598-018-20221-7 (PMC5788866; doi:10.1038/s41598-018-20221-7)
Supplement: Supplementary file 1 — Supplementary Material [file 41598_2018_20221_MOESM1_ESM.pdf]

Supplementary Material

Atypical changes in DRG neuron excitability and complex pain phenotype associated with a $Na_v1.7$ mutation that massively hyperpolarizes activation

Jianying Huang, PhD[1,2], Malgorzata A. Mis, PhD[1,2], Brian Tanaka, PhD[1,2], Talia Adi, BA[1,2] , Mark Estacion, PhD[1,2], Shujun Liu, MS[1,2], Suellen Walker, MBBS, PhD[3], Sulayman D. Dib-Hajj, PhD[1,2], Stephen G. Waxman, MD, PhD[1,2]

[1]Department of Neurology and Center for Neuroscience and Regeneration Research, Yale University School of Medicine, New Haven, CT, USA 06510
[2]Rehabilitation Research Center, Veterans Affairs Connecticut Healthcare System, West Haven, CT, USA 06516
[3]Developmental Neurosciences Program, Department of Anaesthesia and Pain Medicine, UCL Great Ormond Street Hospital, London, WC1N 1EH, UK

Materials and Methods

Plasmids and stable cell lines

The human $Na_v1.7$ cDNA was cloned into a mammalian expression vector and converted into TTX-R phenotype ($hNa_v1.7_R$) by the substitution of tyrosine 362 to serine using site-directed mutagenesis [1]. The $hNa_v1.7_R$/I234T mutation was introduced into $hNa_v1.7_R$ using Quick Change XL site-directed mutagenesis reagents (Stratagene, La Jolla, CA, USA). Wild-type $hNa_v1.7_R$ (referred to as WT hereinafter) or the $hNa_v1.7_R$/I234T (referred to as I234T hereinafter) mutant channels were transfected into HEK293 cells using the Lipofectamine reagent (Invitrogen, Carlsbad, CA, USA). HEK293 cells were maintained in 1:1 Dulbecco's modified Eagle's media (DMEM)/F-12 supplemented with 10% fetal bovine serum in a humidified 5% CO_2 incubator at

37˚C. Transfected HEK293 cells were treated with G418 for several weeks to establish the stable cell lines expressing sodium currents.

Voltage-clamp recordings

Voltage-clamp recordings at room temperature (22 ± 1°C) were performed using an EPC-10 amplifier and the Patchmaster program (v 53; HEKA Elektronik). Recordings at 33˚C were obtained using an Axon MultiClamp 700B amplifier (Molecular Devices, US). Data were digitized via an analogue to digital converter Digidata 1440a (Molecular Devices, US). Acquired data were analyzed using pClamp v10.6, Origin v9.1, Excel and SPSS 24. Temperature was controlled using an adapted Warner Instruments CL-100 temperature controller and SC-20 Dual Inline Heater/Cooler system (Warner Instruments).

Fire-polished electrodes were fabricated from 1.6 mm outer diameter borosilicate glass micropipettes (World Precision Instruments) using a Sutter Instruments P-97 puller and had a resistance of 0.7-1.5 MΩ. Pipette potential was adjusted to zero before seal formation. Liquid junction potential was not corrected. To reduce voltage errors, 80%–90% series resistance compensation was applied. Cells were excluded from analysis if the predicted voltage error exceeded 3 mV. Linear leak currents were subtracted out using the P/N method. Sodium current recordings were initiated after a 5 min equilibration period once whole-cell configuration was achieved. Current traces were sampled at 50 kHz and filtered with a low-pass Bessel setting of 10 kHz. The pipette solution contained the following (in mM): 140 CsF, 10 NaCl, 1 EGTA, and 10 HEPES, 10 dextrose, pH 7.30 with CsOH (adjusted to 310 mOsmol/L with sucrose). The extracellular bath solution contained the following (in mM): 140 NaCl, 3 KCl, 1 $MgCl_2$, 1 $CaCl_2$, 10 HEPES, 10 dextrose, pH 7.30 with NaOH (adjusted to 320 mOsmol/L with sucrose). TTX

(300 nM) was included in the bath to block the endogenous sodium currents in HEK293 cells. Cells were held at -120 mV for all parameters examined. Recovery of $hNa_V1.7$ channels from fast inactivation at room temperature was examined using a two-pulse protocol with interpulse intervals varying from 1 to 513 ms. Recovery rates were measured by normalizing peak current elicited by the test pulse (10 ms depolarization to -10 mV) to that of the prepulse (20 ms at -10 mV) at voltages ranging from -120 mV to -70 mV in 10 mV increments. Normalized peak current at each individual voltage was plotted against the interpulse interval and fit with a single-exponential equation of the form $I = A \times \exp(-t/\tau) + I_c$, where A is the amplitude of the fit, t is time, τ is the time constant of decay, and I_c is the asymptotic minimum to which the tail currents decay. To assess the recovery rate from slow-inactivation at room temperature, cells were prepulsed with a 30 s stimulus at -10 mV to allow the channel to enter slow-inactivation, followed by a range of conditioning voltages from -130 mV to -90 mV in 10 mV increments varying from 4-16384 ms. A 100 ms step to -120 mV was applied immediately afterwards to remove fast inactivation before a 20 ms depolarizing step to -10 mV as a final step to elicit a test response, which reflects the remaining currents that have recovered from slow-inactivation. Sweep interval was increased to 60 s to allow the channel to recover from slow-inactivation.

To assess the biophysical properties of I234T mutant channels at skin/more physiological temperature, we performed voltage-clamp experiments at 33˚C in HEK293 cells stably expressing WT or I234T $Na_V1.7$ channels. Current-voltage relationships were obtained by applying a series of 100 ms depolarizing steps from -80 to +50 mV in 5 mV increments from a holding potential of -120 mV. Conductance was calculated as $G = I / (V_m - E_{Na})$, normalized to the maximum conductance and fit with Boltzmann equation. Steady-state fast inactivation curves were generated by applying 500 ms inactivating potentials from -120 to -10 mV, followed by a

40 ms step to -10 mV. Slow inactivation properties were investigated by stepping the membrane potential from -140 to +10 mV in 10 mV increments for 30 s from V_{hold} of -120 mV, followed by a 100 ms pulse to -120 mV in order to allow recovery from fast inactivation, after which a 40 ms depolarizing step to -10 mV was applied in order to recruit the remaining channels. Normalized conductance values were fit to Boltzmann equation in order to quantify the voltage dependence of steady-state fast and slow inactivation. Deactivation time constants for WT and I234T channels were obtained by fitting single exponential function to tail currents elicited by applying repolarization pulses from -120 to -50 mV after activating the channels at -10 mV for 0.5 ms. Persistent currents were measured as mean amplitudes of currents between 93 and 98 ms after the onset of depolarization, and are presented as a percentage of the maximal peak current.

Independent *t* test was used in statistical analysis unless otherwise mentioned. $P < 0.05$ is considered as statistically significant.

References

1 Herzog, R. I., Cummins, T. R., Ghassemi, F., Dib-Hajj, S. D. & Waxman, S. G. Distinct repriming and closed-state inactivation kinetics of Nav1.6 and Nav1.7 sodium channels in mouse spinal sensory neurons. *J Physiol* 551, 741-750 (2003).

Table S1. Steady-state gating properties of models for WT and I234T $Na_v1.7$ channels in comparison to experimental data.

$hNa_v1.7$	Activation $V_{1/2}$ (mV)	Fast-inactivation $V_{1/2}$ (mV)	Slow-inactivation $V_{1/2}$ (mV)
WT^{exp}	-25.2	-80.3	-64.9
WT^{model}	-24.7	-79.3	-64.7
$I234T^{exp}$	-43.1*	-80.3	-86.2*
$I234T^{model}$	-42.3	-79.3	-87.3

* Statistical significance to WT^{exp} ($p < 0.05$) (Ahn et al., 2010). Exp refers to experimental data from Ahn et al., 2010.

Table S2. Biophysical properties of WT and mutant $Na_v1.7$ channels at 33˚C.

$Na_v1.7$	Activation (mV)			Steady-state fast inactivation (mV)			Steady-state slow inactivation (mV)			Ramp current			Persistent current	
	$V_{1/2,act}$	k	n	$V_{1/2,fast}$	k	n	$V_{½,slow}$	k	n	% of I_{peak}	V_{peak} (mV)	n	% of I_{peak}	n
**WT**	-20.2±1	4.9±0.3	21	-69±1	5.9±0.1	19	-77.1±2	8.7±0.9	13	0.66±0.2	-40.9±3	14	2.9±0.4	19
**I234T**	-34±1***	5.8±0.2*	25	-67.6±2	5.5±0.2	15	-87.2±2***	5.2±0.2**	15	3.1±0.3***	-49±2*	16	4.6±0.6*	22
**ΔV**	-14 mV			No change			-10 mV				-8 mV		N/A	

* p<0.05, ** p<0.01, *** p<0.001 versus WT channels

Figure Legends

Figure S1. Recovery rates from (A) fast-inactivation and (B) slow-inactivation for WT and I234T hNa$_v$1.7 channel. The protocol for recovery from slow-inactivation was illustrated in the lower panel of B.

Figure S2. Kinetic models of WT and I234T hNa$_v$1.7 channel based on Hodgkin-Huxley equations. (A) Voltage-dependence of conductance/maximal conductance (G/G$_{max}$) at steady-state for activation (*m*), fast-inactivation (*h*) and slow-inactivation (*s*). Solid lines represent WT channel and dash lines represent I234T channel. Voltage dependencies of time constants for **(B)** activation and deactivation, **(C)** fast-inactivation and repriming, and **(D)** slow-inactivation and recovery from slow-inactivation.

Figure S3. Biophysical properties of WT and I234T channels at 33°C. (A) Normalized peak current-voltage relationship for WT and I234T channels. **(B)** Voltage-dependence of activation for WT and I234T channels. The conductance curves were obtained by Boltzmann fits to the normalized conductance. The activation curve for I234T channels is shifted towards more hyperpolarized potentials ($p < 0.001$). **(C)** Representative ramp currents from WT and I234T channels induced by 150 ms depolarizations from -120 to 0 mV, presented as a percentage to the peak currents obtained from the current-voltage relationships. **(D)** I234T channels produced significantly larger ramp currents than WT ($p < 0.001$). Each symbol represents an individual cell. Black lines among the symbols indicate average amplitudes of ramp currents. **(E)** Deactivation time constants for WT and I234T channels ($F = 38$, $p < 0.001$; one-way repeated measures ANOVA). Time constants for deactivation were obtained by single exponential fits to

the tail currents. **(F)** Steady-state fast-inactivation for WT and I234T channels. Boltzmann fits to inactivation curves did not reveal a significant difference in $V_{½}$ between WT and I234T channels ($p = 0.5$). **(G)** Steady-state slow-inactivation for WT and I234T channels. The hyperpolarized shift in I234T slow-inactivation was significant ($p < 0.001$). **(H)** Late **(**persistent) current, presented as percentage of peak current, was significantly increased in I234T mutant channel as compared to WT ($p < 0.05$). **(I)** Representative trace demonstrating late (persistent) current of WT channel. **(J)** Representative trace demonstrating late (persistent) current of I234T mutant channel.

Figure S1

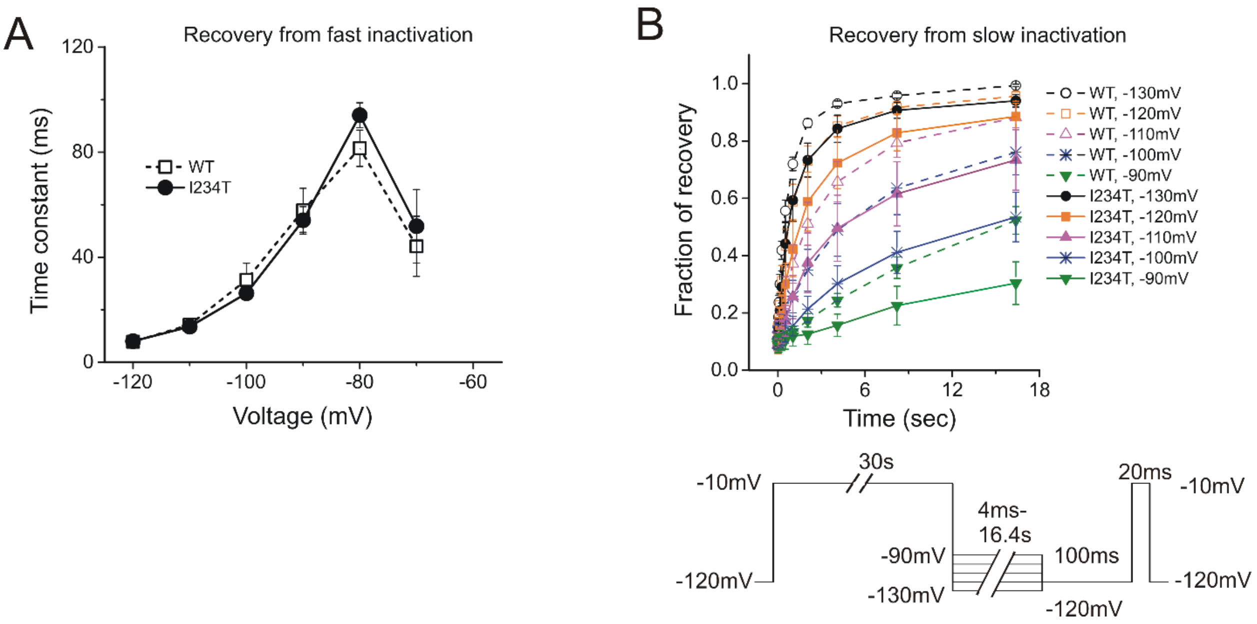
A
Recovery from fast inactivation
Time constant (ms)
Voltage (mV)
WT
I234T
B
Recovery from slow inactivation
Fraction of recovery
Time (sec)
WT, -130mV
WT, -120mV
WT, -110mV
WT, -100mV
WT, -90mV
I234T, -130mV
I234T, -120mV
I234T, -110mV
I234T, -100mV
I234T, -90mV
-10mV
30s
-120mV
4ms-
16.4s
-90mV
-130mV
100ms
-120mV
20ms
-10mV
-120mV

Figure S2

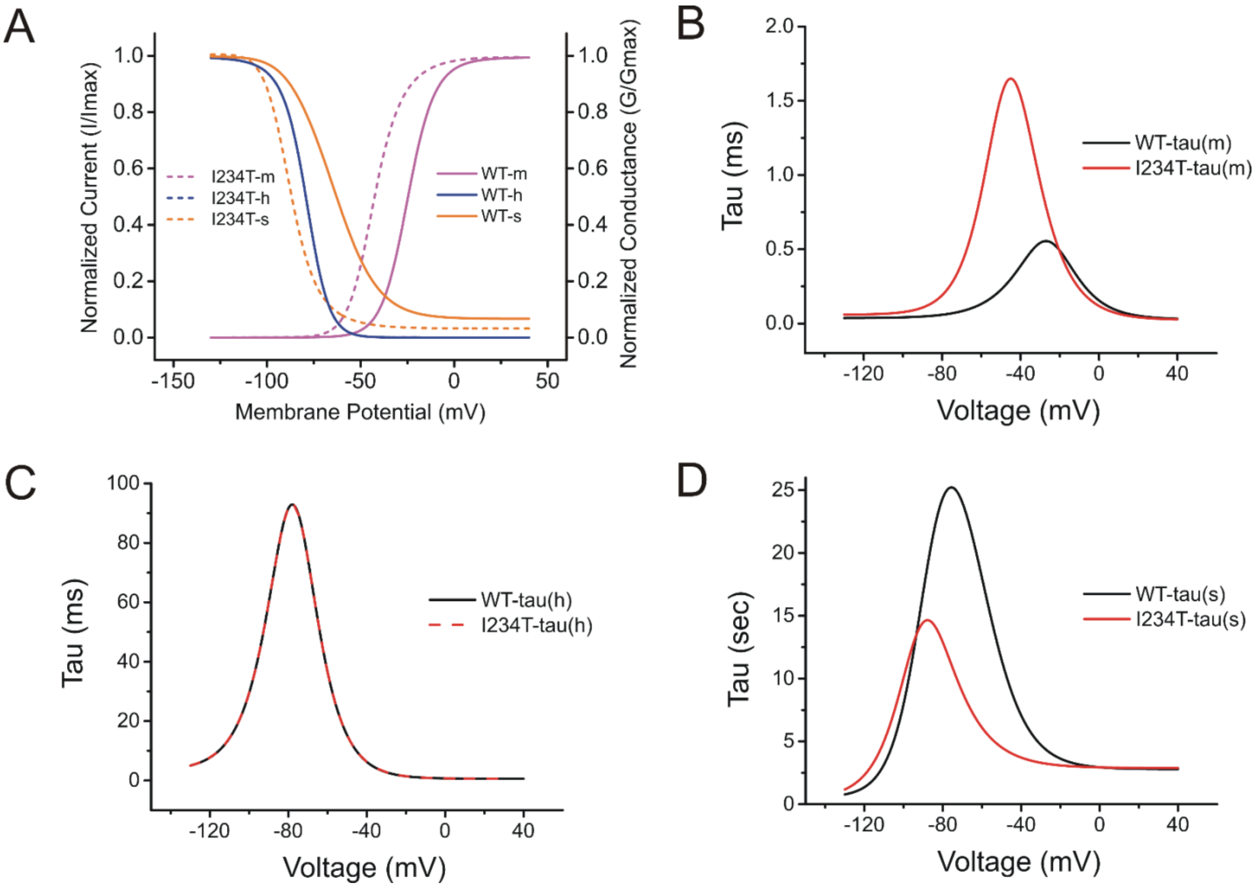
A
Normalized Current (I/Imax)
Normalized Conductance (G/Gmax)
Membrane Potential (mV)
I234T-m
I234T-h
I234T-s
WT-m
WT-h
WT-s
B
Tau (ms)
Voltage (mV)
WT-tau(m)
I234T-tau(m)
C
Tau (ms)
Voltage (mV)
WT-tau(h)
I234T-tau(h)
D
Tau (sec)
Voltage (mV)
WT-tau(s)
I234T-tau(s)

Figure S3

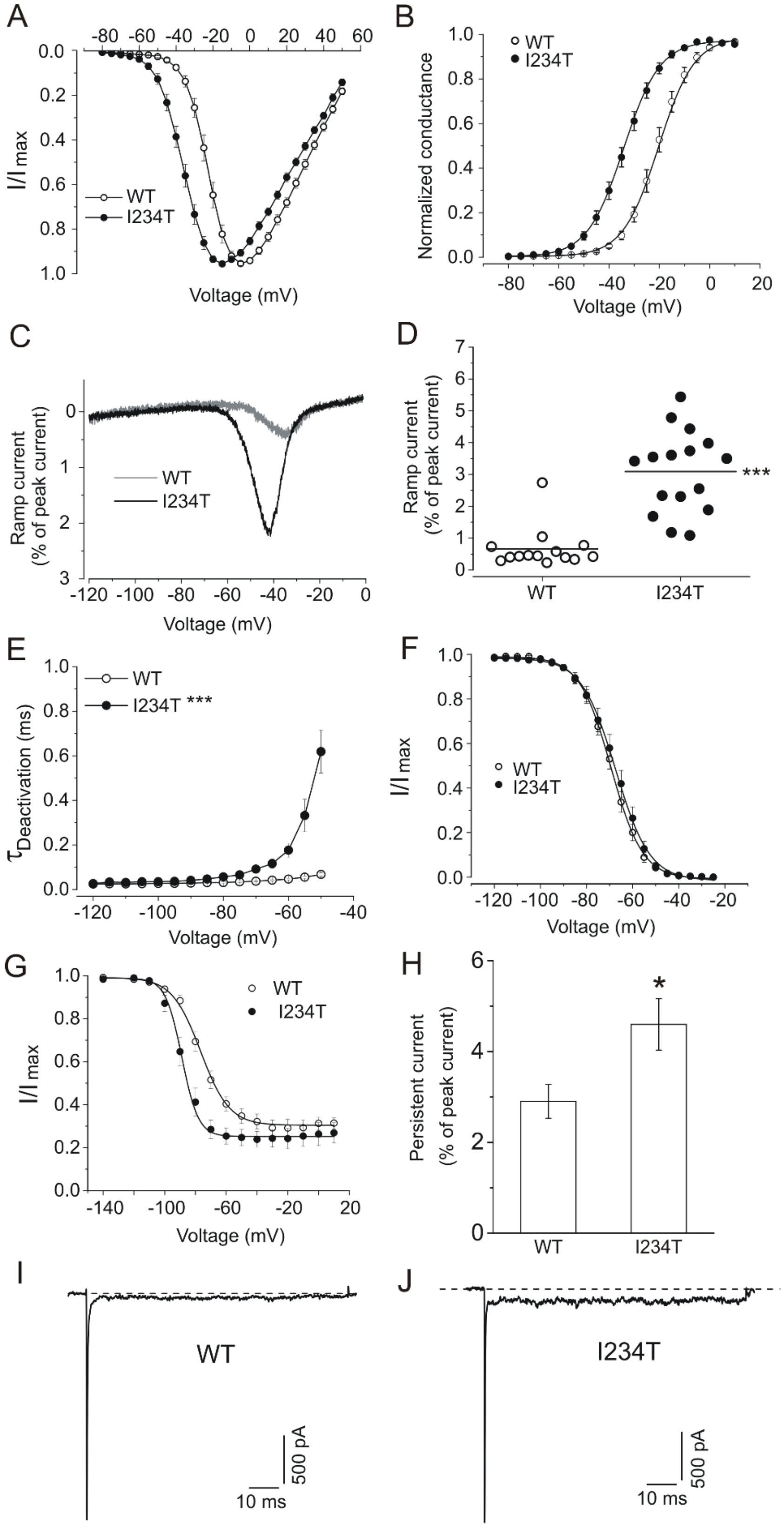
A
-80 -60 -40 -20 0 20 40 60
0.0
0.2
0.4
0.6
0.8
1.0
I/I max
WT
I234T
Voltage (mV)
B
1.0
0.8
0.6
0.4
0.2
0.0
Normalized conductance
WT
I234T
-80 -60 -40 -20 0 20
Voltage (mV)
C
Ramp current
(% of peak current)
0
1
2
3
WT
I234T
-120 -100 -80 -60 -40 -20 0
Voltage (mV)
D
7
6
5
4
3
2
1
0
Ramp current
(% of peak current)
***
WT
I234T
E
1.0
0.8
0.6
0.4
0.2
0.0
τDeactivation (ms)
WT
I234T ***
-120 -100 -80 -60 -40
Voltage (mV)
F
1.0
0.8
0.6
0.4
0.2
0.0
I/I max
WT
I234T
-120 -100 -80 -60 -40 -20
Voltage (mV)
G
1.0
0.8
0.6
0.4
0.2
0.0
I/I max
WT
I234T
-140 -100 -60 -20 20
Voltage (mV)
H
6
4
2
0
Persistent current
(% of peak current)
*
WT
I234T
I
WT
500 pA
10 ms
J
I234T
500 pA
10 ms
